# Development and User Experience Evaluation of an Experience Sampling-Based Dietary Assessment Method

**DOI:** 10.1016/j.cdnut.2024.104479

**Published:** 2024-10-15

**Authors:** Joke Verbeke, Christophe Matthys

**Affiliations:** 1 Department of Chronic Diseases and Metabolism, Clinical and Experimental Endocrinology, KU Leuven, Leuven, Belgium; 2 Department of Endocrinology, University Hospitals Leuven, Leuven, Belgium

**Keywords:** ecological momentary assessment, nutrition assessment, mobile, digital health, digital biomarker, epidemiology

## Abstract

**Background:**

Most technology-based dietary assessment methods use the same methodology as traditional dietary assessment methods resulting in similar limitations and biases. Experience sampling methodology (ESM) is a real-life real-time data-capturing method that is explored as an alternative methodology for dietary assessment to improve feasibility and data accuracy.

**Objectives:**

This research aimed to develop and evaluate an experience sampling-based dietary assessment method (ESDAM) measuring habitual dietary intake.

**Methods:**

Starting from a food frequency questionnaire, experience sampling principles were implemented resulting in a pilot ESDAM. Second, the pilot ESDAM was evaluated for feasibility and convergent validity compared with a 3-d food record. Mean intake with standard deviations was compared between the pilot ESDAM, food record (FR), and food frequency questionnaire (FFQ), and Spearman correlation coefficients (SCCs) were calculated. Third, following a literature review and expert opinion, the questions and design of the pilot ESDAM were further adapted to ESM and implemented in an experience sampling survey application. The resulting prototype ESDAM underwent 2 rounds of user experience (UX) evaluation in which 10 persons tested ESDAM for 1 wk followed by a structured evaluation interview.

**Results:**

The pilot ESDAM, FR, and FFQ were completed by 27 participants and the evaluation questionnaire by 78 participants. Mean energy intake by the FFQ, pilot ESDAM, and FR was 1272.2 ± 308.9 kcal/d, 1592.3 ± 358.9 kcal/d, and 1664.6 ± 257.8 kcal, respectively. The evaluation revealed the limited time window (19:00–23:00) to respond was inconvenient, good acceptability, and ease of use of the pilot ESDAM. The UX evaluation study revealed overall good acceptability, ease of use, and low burden of the different prototypes of ESDAM.

**Conclusions:**

ESM could advance the field beyond traditional methodologies and improve feasibility. ESDAM is unique in assessing dietary intake quantitatively through ESM. Additional assessment of validity might shed light on the data accuracy of ESDAM.

## Introduction

Measurement errors in dietary intake data challenge the field of nutrition research, ultimately impeding the advancement of nutrition-health research [1,2]. A high burden to register details on food intake for longer periods, reporting fatigue, social-desirability bias, recall bias, and misreporting are well-known limitations in self-reported dietary assessment contributing to measurement errors that undermine the quality of dietary intake data [3]. True dietary intake cannot be measured, and a complete panel of dietary biomarkers to objectively assess dietary intake is currently not available [4]. Hence, it is imperative to further research and develop self-reported dietary assessment methods that minimize biases and reduce measurement errors to advance nutrition research.

Web-based or app-based versions of food records, food frequency questionnaires (FFQs), or 24-hour dietary recalls (24HRs) are a significant step forward in terms of feasibility (i.e., ease of use, remote data collection, automatic nutrient calculation) for both respondent and researcher [5]. However, significant measurement errors remain even in the Web-based and app-based versions of traditional dietary assessment methods [6,7]. Shifting focus toward new methodologies, in addition to new technologies, to enhance the feasibility of dietary assessment methods could pave the way for new advancements. Methodologies that enhance feasibility by easy implementation in daily life, ease of use, minimum time consumption, and require minimum brainpower might reduce burden, reporting fatigue, social-desirability bias, recall bias, misreporting, and, thus measurement errors by its design. Moreover, feasibility was shown to be the main criterion for selecting a dietary assessment method by researchers [8].

Still, new methodologies, apart from new technologies, for dietary assessment remain largely unexplored [9,10].

Therefore, new methodologies for dietary assessment leveraged by technology that simultaneously maximizes feasibility and accuracy of dietary intake data are a focus point in researching and developing new dietary assessment methods.

Experience sampling methodology (ESM), used interchangeably with ecological momentary assessment (EMA), could be a promising alternative methodology for the field of dietary assessment. Originating from psychology and behavioral sciences, ESM involves the technique of requesting a person to report in the moment itself in daily life on, e.g., emotions or behaviors by mobile prompt messages [[11], [12], [13]]. A few short questions, sent at random moments during the day, allow capturing real-life momentary data reducing recall bias, reactivity bias, and misreporting in psychology and behavioral research [14]. The strength of ESM lies in the real-time monitoring by smartphone prompts with short easy-to-answer questions. ESM is specifically developed to maximize the feasibility for the respondents to obtain more qualitative data. The ease of use can lower the burden for respondents allowing for longer measurement periods. Near–real-time measurements can reduce recall bias, whereas the unannounced assessments may reduce social-desirability bias. Therefore, ESM is promising as a dietary assessment methodology in the search toward a feasible method that yields qualitative data. In addition, ESM could be used to assess environmental or psychological factors alongside dietary intake to obtain more insight into determinants of dietary intake, for example.

However, little research has been done on the application of ESM as an alternative dietary assessment method to traditional dietary assessment methods such as FRs, 24HRs, and FFQs. Schembre et al. [14] conducted a systematic review of diet assessment methods based on EMA for behavioral research. In addition, König et al. [15] identified dietary assessment methods based on EMA in a systematic review of smartphone-based dietary assessment tools. However, most dietary assessment methods described in the aforementioned reviews do not use ESM or EMA as an alternative dietary assessment method (i.e., to assess dietary intake quantitatively such as nutrient or food group intake). Instead, most dietary assessment methods described in literature stem from behavioral research and assess eating behaviors (i.e., eating episodes without specification of type or quantity of foods consumed, cravings, binge eating episodes) rather than dietary intake (i.e., quantification of nutrients and food group intake) [16]. As a result, EMA-based dietary assessment methods in the literature are mostly intended to describe dietary intake qualitatively (i.e., type of foods, time of eating episode). Based on current literature, Jeffers et al. [17] and Lucassen et al. [18,19] were the first to develop a FR based on EMA with the same purpose as traditional dietary assessment methods (i.e., quantification of nutrient and food group consumption, diet quality). This manuscript describes the stepwise development of an experience sampling-based dietary assessment method (ESDAM) followed by user experience (UX) testing. ESDAM is developed as an alternative method to traditional dietary assessment methods to assess the habitual dietary intake of the Flemish (Belgian) adult population.

## Methods

**FIGURE 1:**
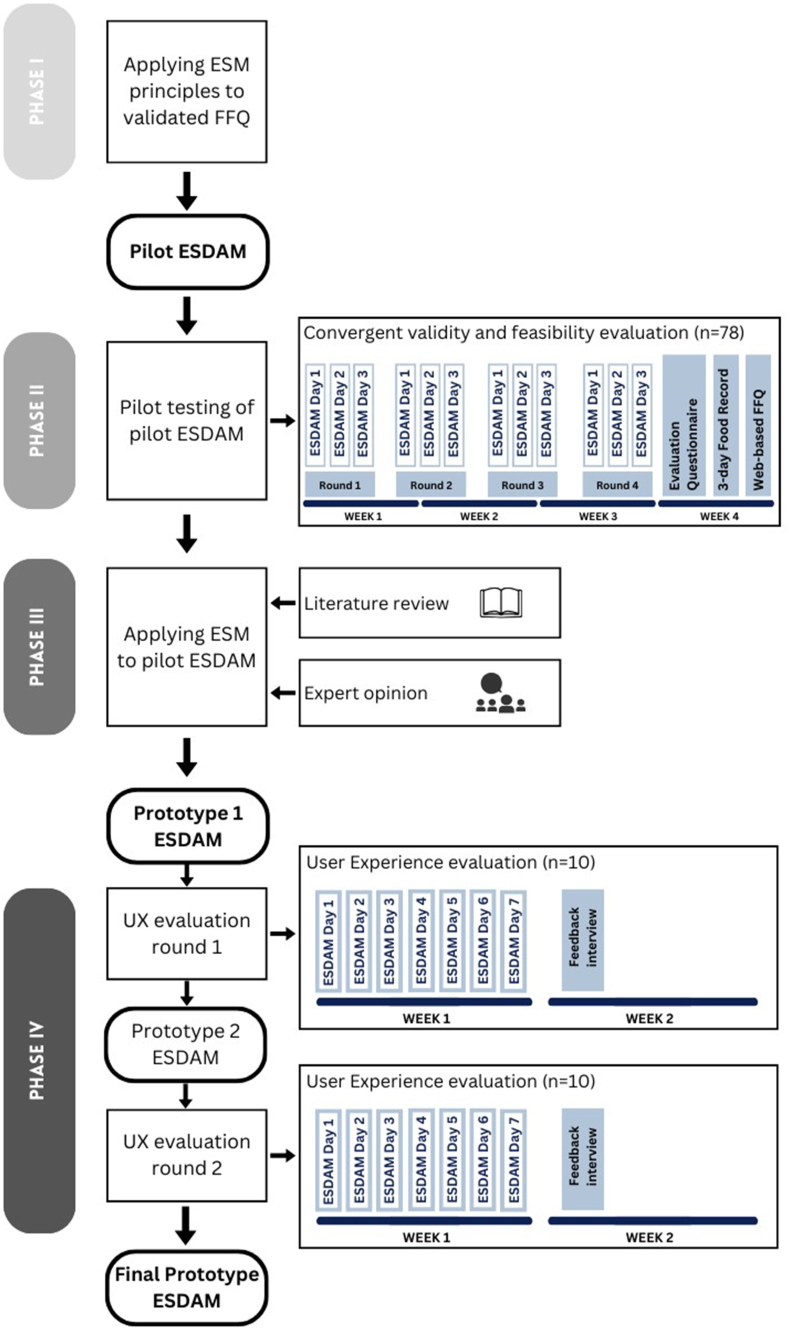
Iterative development and evaluation process of the experience sampling-based dietary assessment method (ESDAM). UX, user experience.

The ESDAM was developed following an iterative approach as presented in Figure 1. In summary, first principles of experience sampling were applied to a validated 32-item Web-based FFQ, described elsewhere, that was developed to assess the habitual dietary intake of the Flemish (Belgian) population [20]. This resulted in the pilot version of ESDAM. The pilot version of ESDAM was developed and evaluated initially to assess the potential of ESM to increase the feasibility of dietary assessment before performing in-depth research on the development of an ESDAM. Additionally, no literature was available on how to apply ESM for dietary assessment specifically. Second, the pilot version was tested by evaluating convergent validity and feasibility. Third, experience sampling principles were applied further (i.e., ESM sampling schedule) to the pilot ESDAM resulting in the first prototype of the ESDAM. Lastly, the prototype of the ESDAM was tested for UX (i.e., usability and feasibility) and adapted until the final version of the ESDAM was obtained.

### Phase I: Development of pilot version based on FFQ

**FIGURE 2:**
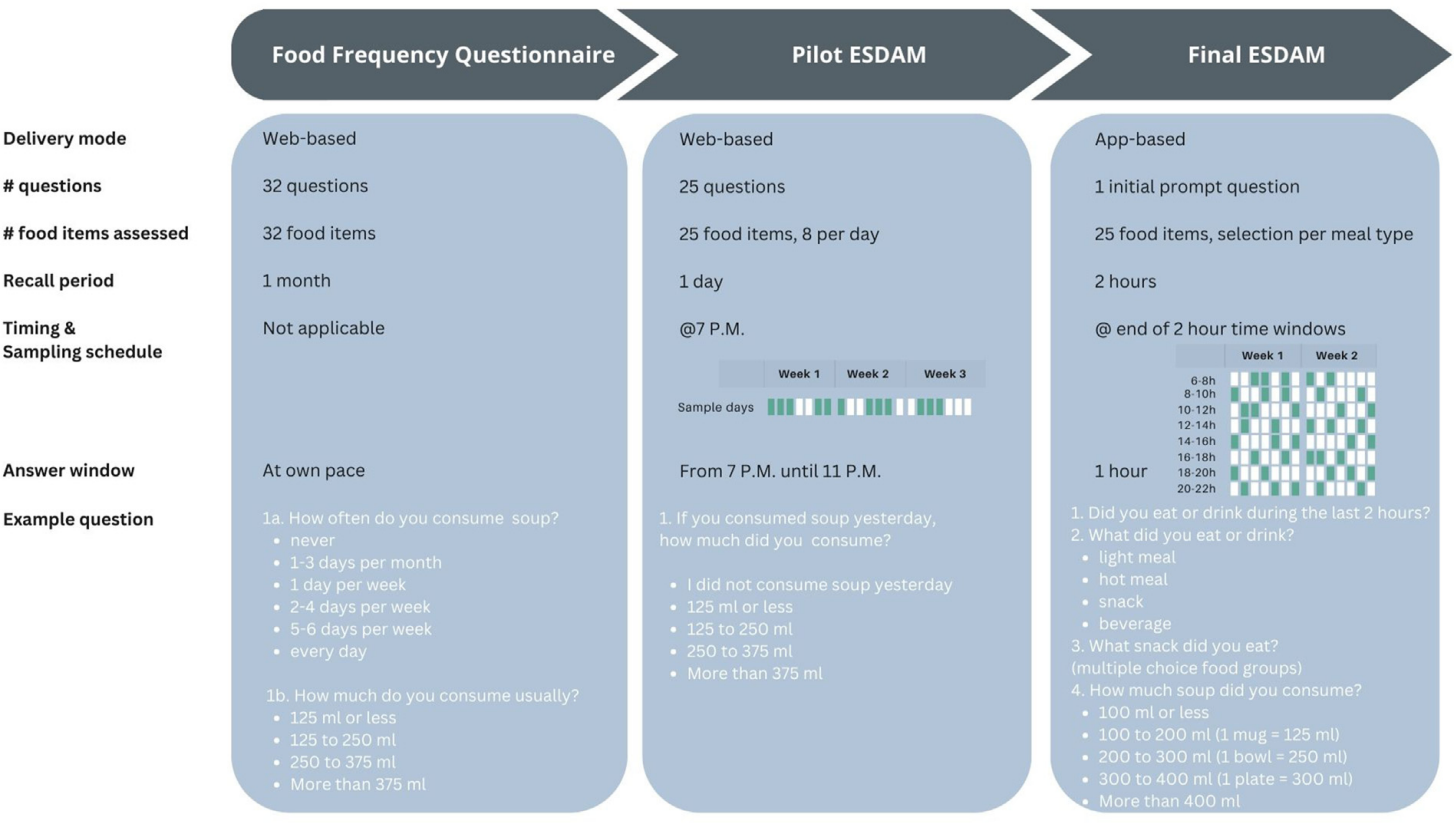
Characteristics and formulation of questions of the food frequency questionnaire compared with the pilot experience sampling-based dietary assessment method (ESDAM) and final ESDAM.P.M., postmeridiem.

In phase I, it was aimed to apply principles of the ESM to a validated Web-based FFQ to explore the feasibility of ESM as a dietary assessment method and gain first insights on data quality [20,21]. The Web-based FFQ was validated against 3-d food records, which showed that the FFQ underestimates absolute nutrient intake and intake of most food groups [20]. The focus in this first step was on the reduction of the recall period and length of the questionnaire to reduce the burden and improve feasibility, in line with the ESM guidelines. As a first step, the 32 questions of the FFQ assessing the consumption of 32 food groups during the past month were regrouped into 25 food groups. A key principle of ESM is to minimize the time needed to complete the questions in the prompt. Therefore, it is important to optimize the flow of the questions so that limited screens need to be passed by the respondent. Therefore, some of the 32 food groups of the FFQ, described elsewhere, were merged into broader categories (i.e., white bread and wholegrain bread merged into the category “bread”) with an additional subquestion prompted in the next screen (i.e., to indicate if it was white or wholegrain bread, only shown to those that indicated consumption of bread) [20]. In this way the questionnaire for the ESM became branched, meaning that subquestions were shown only for those respondents who checked certain response options (e.g., subquestion to specify the type of bread is only presented to those that indicated consumption of bread). By doing so, fewer food groups and, thus, multiple choice response options, needed to be presented initially on the screen, and the minimum set of questions, and thus screens, could be presented for each respondent. Next, the questions of the FFQ were reformulated so that the recall period was reduced from the past month to the previous day as shown in Figure 2. Finally, the 25 questions on the consumption of 25 food groups were divided at random over 3 consecutive days. As a result, the pilot ESDAM assessed the consumption of ∼8 food groups of the previous day during 3 consecutive assessment days. To ensure data quality, the pilot ESDAM was repeated 4 times with 2 days in between each round during a period of 3 wk as represented in Figure 2. In this way, the consumption of every food group is assessed 4 times in total. The questions were sent to the study participants (*n* = 99) through a Web-based questionnaire platform (Qualtrics) via email at 19:00 Typically in ESM, the questionnaire is available to complete for a limited time only (i.e., 1 h) after receiving the prompt message that asks to complete a new questionnaire. To implement this characteristic of ESM, participants were informed that the questionnaire was available to complete from 19:00 until 23:00. This time window was chosen arbitrarily as a first test, the link to the questionnaire did not work anymore after 23:00.

### Phase II: Pilot testing

In Phase II, the pilot ESDAM was tested for feasibility and convergent validity as demonstrated in Figure 1. Feasibility was evaluated through an evaluation questionnaire adapted from the mobile Health (mHealth) App Usability Questionnaire [22]. The evaluation questionnaire was sent to the study participants (*n* = 99) through email using a Web-based questionnaire platform (Qualtrics) after the completion of 4 rounds of the pilot ESDAM (Figure 1). Convergent validity for absolute and relative macronutrient intake was evaluated by assessing correlations between the pilot ESDAM with a 3-day food record using the MyFitnessPal application and the Web-based FFQ [20]. Anonymized study accounts were created in MyFitnessPal by the researchers and provided to the participants to register the type and quantity of foods during 3 nonconsecutive days. The food record included ≥1 d of the weekend. Log reports of the study accounts in MyFitnessPal were extracted to derive the type and quantities of foods consumed (not nutrient intake). Daily energy and nutrient intake of the food records and pilot ESDAM were calculated based on the Belgian Food Composition Table [23]. A sociodemographic questionnaire assessed age, gender, educational level, employment status, length, and (self-reported) body weight. This study was approved by the Social and Societal Ethics Committee (SMEC) review board (G-2021-2956). All participants provided their written informed consent before the start of the study.

It was aimed to recruit 100 participants following recommendations of Willet et al. [24] to have a sample size of a minimum of 50 participants, preferably ≥100, for validation of dietary assessment methods. Recruitment took place between February 2021 and April 2021 through announcements on social media. Participants were eligible if they were 25 y or older, did not follow a diet for medical reasons (i.e., celiac disease), and had a smartphone. Participants with insufficient knowledge of Dutch to complete the questionnaires were excluded.

### Phase III: Development prototype ESDAM

The prototype was developed based on a literature review combined with expert opinion as described below.

#### Literature review to develop sampling scheme of ESDAM

In Phase III, it was aimed to apply the ESM further including an ESM sampling scheme (i.e., multiple random prompts with questions within 1 d). However, defining and balancing ESM characteristics, i.e., study duration, frequency and timing of sampling, formulation of questions, and response options, is a delicate matter and crucial to obtaining accurate data and good feasibility using ESM [25]. Therefore, a literature review was conducted to explore how ESM could be applied to dietary assessment as described elsewhere [16]. The literature review concludes that ESM could be applied for dietary assessment following a FR approach to measure actual dietary intake or based on an existing FFQ to measure habitual dietary intake with both approaches needing different durations, sampling schemes, and questionnaire designs. An example of the implementation of ESM following a FR approach or based on an FFQ is described elsewhere [16].

Because it was aimed to develop a dietary assessment method based on ESM to measure habitual dietary intake, we built further on the pilot ESDAM by adapting these questions to suit the typical ESM format and developed a sampling scheme suited for measuring habitual dietary intake. The typical ESM format includes an initial question shown in the prompt message, sent by the ESM questionnaire application, followed by more in-depth questions. Moreover, the questions and response options were developed according to the recommendations described by Eisele et al. [21] on the development of ESM questionnaires. In short, ESM questionnaires are designed with the main focus to disturb the respondent as little as possible in daily life to obtain more accurate data with less bias. Therefore, questions and response items are developed to be as short as possible, to-the-point, using unambiguous wording. Questions should be ordered and formulated to obtain an optimal flow of questions so that the respondent is bothered with as few screens as possible to pass to complete the questionnaire in the app. ESM questionnaires are recommended to take 2 min or less to complete, therefore questionnaire length should be limited.

Following the recommendations described in the literature review, a sampling scheme was created with 3 prompts per day at fixed times at the end of 2-h windows between 06:00 and 22:00 for a duration of 2 wk as shown in Figure 2 [16]. Every prompt asked to report dietary intake during the past 2 h. A recall period of 2 h was chosen to reduce the recall period and burden further in compliance with ESM design recommendations and this being the most used recall period in ESM according to the literature review. The sampling scheme was composed so that every 2-h time window between 06:00 and 22:00 was sampled 5 times within the 2 wk of the ESM sampling schedule.

#### Expert opinion to design questionnaire for ESDAM

Expert’s opinion (*n* = 4) was consulted by following the think-aloud protocol in a group discussion to formulate response options to the ESDAM prompt, which asked to report dietary intake during the past 2 h. Experts were recruited by convenience sampling within the research department and had a background in dietetics and research related to health and nutrition. It was aimed to reformulate and regroup the food groups of the pilot ESDAM (originating from the FFQ) further so that the amount of multiple choice response options and screens to pass in the ESM app were reduced to minimize the burden and time needed to respond as advised by ESM design recommendations [21]. The ESDAM questionnaire was developed aiming at a total response time of 2 min at maximum. Additionally, the questionnaire of the ESDAM was designed for and adapted to the dietary habits of the target population (i.e., Flemish (Belgian) adults).

#### Design of the prototype ESDAM

**FIGURE 3:**
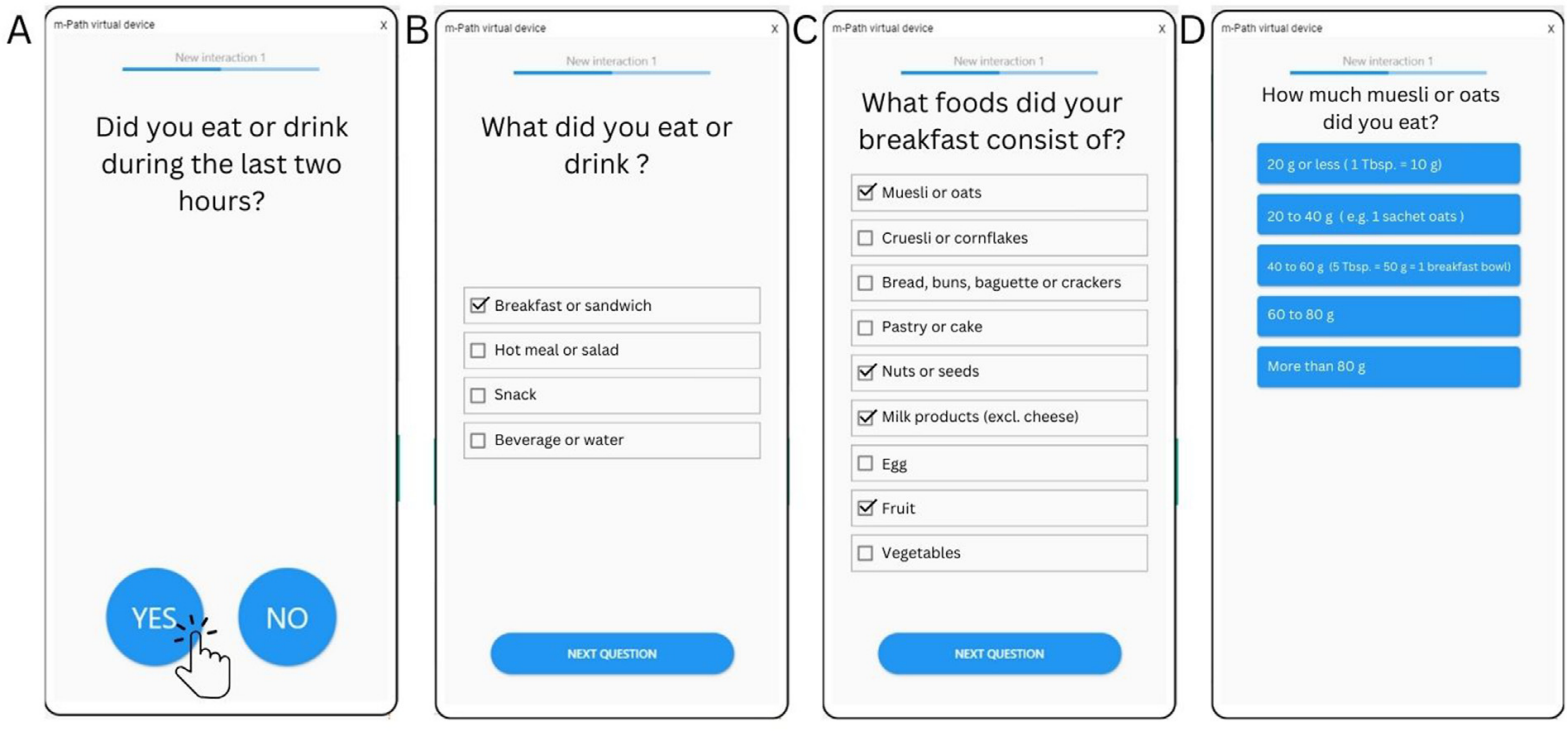
Presentation of initial screens (A–D) with questions of the prototype ESDAM embedded in the ESM app (mPath). ESDAM, experience sampling-based dietary assessment method; ESM, experience sampling method.

Based on the expert’s consensus, the initial prompt was formulated as “Did you eat or drink something during the past 2 h?” with a “yes” or “no” response option. Next, it was agreed upon to let multiple choice response options allow to indicate the type of meal (beverage or water, snack, breakfast or sandwich, main meal or salad) in case of a positive response to the initial prompt. Based on the selected meal type, multiple choice options of a selection of food groups (originating from the pilot ESDAM) allow to indicate which food groups were consumed. This approach was chosen to limit the amount of screens to pass in the ESM application and limit the time needed to respond in line with ESM guidelines. Figure 3 shows the initial screens with questions of the prototype ESDAM. The selection of food groups shown as multiple choice response options following each of the different meal types were selected based on the agreement between the experts and based on the Belgian dietary pattern. Lastly, multiple choice response options to indicate the portion size consumed by each food group in the selected meal were adapted from the pilot ESDAM (originating from the FFQ that was developed based on national food consumption survey data).

Therefore, the original response options of the pilot ESDAM and FFQ to indicate portion sizes were revised based on prior data of the validation of the FFQ (*n* = 100) [20]. The answer options for the portion size of each food group of the FFQ were plotted as a distribution and visually inspected for normality or skewness. Skewness to the left (i.e., most responses indicate consumption of small portion sizes) was interpreted as current response options for portion size being too large. However, skewness to the right (i.e., most responses indicate consumption of large portion sizes) was interpreted as current response options for portion size being too small. As a result, 5 instead of the original 4 multiple choice response options for portion size were created either by adapting current response options to smaller variance of portion size within each response option or, in case of skewness of distribution to the right, adding an extra response option with larger portion size. To aid in the estimation of portion sizes, the response options to indicate portion size included examples of portion sizes in household measures of foods typically consumed within that food group. The prototype 1 ESDAM sampling schedule and questionnaire were implemented in an ESM questionnaire app (mPath) [26].

### Phase IV: UX evaluation of prototype ESDAM

The last step includes the UX testing of the prototype ESDAM (Figure 1). This study aimed to evaluate the UX and feasibility of the prototypes of ESDAM in potential end-users to optimize the frequency and timing of prompts together with the formulation of questions and response options. Therefore, the prototype ESDAM was tested by participants for 1 wk followed by a structured interview to assess the UX. This test and evaluation were repeated with optimization of the prototypes of ESDAM in between each test round, including 10 participants each, until data saturation was reached. A sample size of 10 participants was shown to be able to identify ≥ 80% of issues in usability testing, whereas 20 participants could identify ≥ 95% of issues in usability tests [27]. Participants (*n* = 20) were recruited through calls on social media and within the university campus between December 2023 and February 2024. Participants were eligible if they had a smartphone, were ≥18 y old, and had sufficient knowledge of the Dutch language to complete the study. No exclusion criteria were applicable. At the start of the study, all participants received instructions to download and install the mPath application in which the prototypes of ESDAM were embedded. A short explanation of the study and an introduction to the use of the prototype ESDAM were given to participants before the start of the ESM sampling. Next, participants received for 7 consecutive days 3 times a day the ESDAM prompt to indicate if anything was eaten or drunk during the past 2 hours followed by multiple choice response options as described above. Lastly, all participants were contacted for a telephone or in-person structured interview to assess the UX and feasibility of the prototype ESDAM. The interview assessed app experience, ease of use, flow, burden (time consumption), timing and frequency of prompts, content of questions and response options, and comparison with previously used diet tracking applications. Data on age and gender of participants were collected during the interview. The interviews were analyzed through thematic content analysis. Following the feedback of the first UX evaluation round, the first prototype ESDAM was adapted and resulted in the second prototype ESDAM, which was UX evaluated following the same procedure (Figure 1). All participants gave informed consent to participate before the start of the study. This study was approved by the SMEC review board (G-2022-5763-R2(MIN)).

### Data analysis

The convergent validity of the pilot ESDAM was evaluated through a comparison of mean intake with standard deviations (SDs) between the ESM, FR, and FFQ for energy and macronutrients. Mean difference, Wilcoxon signed rank test, and Spearman correlation coefficients (SCCs) were calculated. Only participants with complete FFQs, who completed ≥3 of 4 pilot ESDAM rounds and with food diaries consisting of 3 d with ≥2 main meals (breakfast, lunch, or dinner) registered daily were included for analysis of convergent validity of the pilot ESDAM. Spearman's correlation coefficients were interpreted according to Cohen's cutoff values, where *r* = ±0.5 was considered to be strong, *r* = ±0.30 moderate, and *r* = ±0.10 weak [28]. *t* Tests were used to evaluate statistically significant differences for discrete variables of the UX evaluation questionnaire and sociodemographic data whereas the chi-square test was performed for dichotomous variables. A *P* value of <0.05 indicated statistical significance. The statistical program IBM SPSS Statistics 27 was used for all analyses.

### Sample size and power analysis

Post hoc power analysis was performed for the pilot ESDAM evaluation against food records and the FFQ including 27 participants. G-Power 3.1.9.7 was used for power calculations to detect correlation coefficients of 0.30 and 0.50 for an alfa error probability of 0.05 (2-tailed) in a sample of 27 participants.

## Results

### Validity and feasibility of the pilot ESDAM

A total of 99 participants completed ≥1 round of the pilot ESDAM. Furthermore, 81 (81.8%) participants completed ≥2 rounds, 55 (55.6%) participants ≥3 rounds, and 23 participants (23.2%) completed all 4 rounds. The FFQ was completed by 91 participants, and the 3-d food record was completed by 45 participants. The pilot ESDAM was completed for ≥3 of 4 rounds together with the FFQ and a 3-d food record (FR) by 27 participants. Of the 27 participants who completed both the pilot ESDAM for ≥3 rounds together with the FR and FFQ, 26 participants filled out the sociodemographic questionnaire. Sociodemographic data were available for 96 participants out of the 99 participants in the pilot study. Table 1 presents sociodemographic data of participants included and excluded in the convergent validity analysis of the pilot ESDAM. The study population of the pilot test consisted mainly of highly educated women (88%) with a mean (SD) age of 40 (±14) y.

**TABLE 1:** Sociodemographic characteristics of participants included in the convergent validity analysis of the pilot ESDAM compared with participants that were not eligible and excluded from the analysis.

	Included	Excluded	Statistical difference *P* value
Participants with sociodem. data	*N* = 27	*N* = 72	
Missing sociodem. data	*N* = 1	*N* = 2	
Gender			0.106
Women	23 (89%)	51 (73%)	
Men	3 (11%)	19 (27%)	
Educational level			0.384
Secondary educational level	0 (0%)	2 (3%)	
Higher educational level	26 (100%)	68 (97%)	

Age (mean ± SD)	40 ± 14	43 ± 14 y	0.434

Anthropometrics			
Height (mean ± SD)	169 ± 8 cm	171 ± 8 cm	0.171
Weight (mean ± SD)	68 ± 12 kg	72 ± 13 kg	0.188
Body mass index (mean ± SD)	24 ± 3 kg/m^2^	24 ± 4 kg/m^2^	0.405

Abbreviation: ESDAM, experience sampling-based dietary assessment method.

^1^sociodem. = sociodemographic.

**FIGURE 4:**
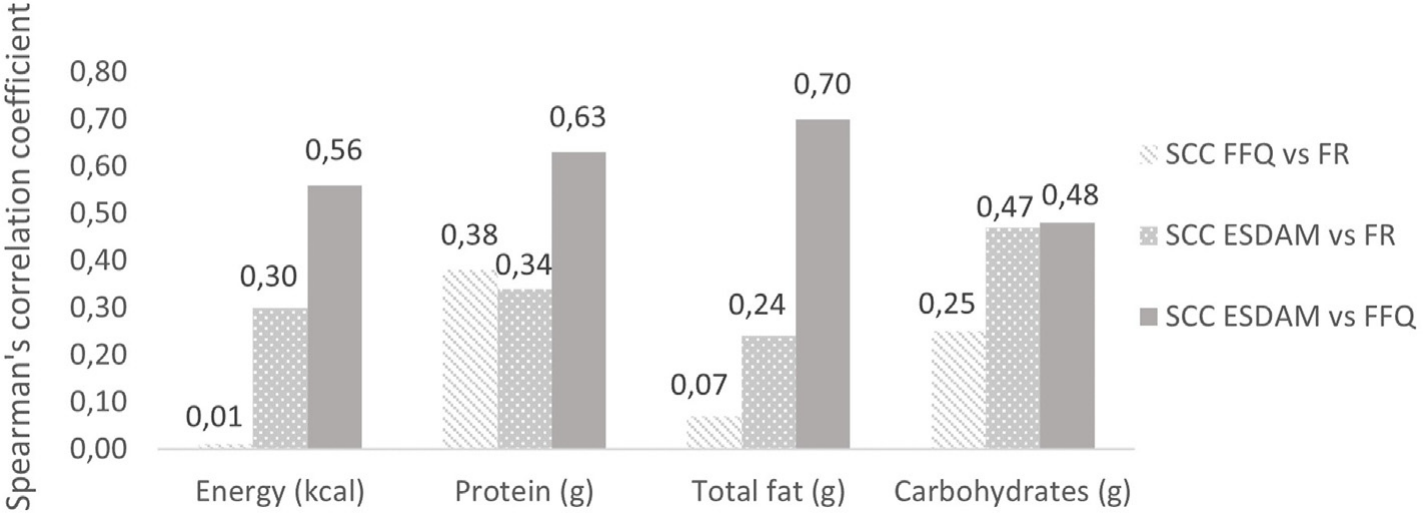
Correlation coefficients for energy and macronutrients between FFQ and FR, pilot ESDAM and FR, pilot ESDAM and FFQ, respectively. ESDAM, experience sampling-based dietary assessment method; FFQ, food frequency questionnaire; FR, food record; SCC, Spearman correlation coefficient.

The mean intake of energy and macronutrients obtained through the FFQ, pilot ESDAM, and FR are shown in Table 2 together with convergent validity parameters of the pilot ESDAM compared with the FR. SCCs were lowest for absolute fat intake and relative protein intake. The highest correlations were found for both absolute and relative carbohydrate and relative fat intake (Table 2). The relative mean difference in intake for all macronutrients was 2% or lower and not statistically significant (Table 2). Figure 3 shows SCCs for energy and macronutrients between the pilot ESDAM and the FFQ, the pilot ESDAM, and the FR, and between the FFQ and the FR. Correlation coefficients are highest for energy and all macronutrients between the FFQ and the pilot ESDAM. Figure 4 presents the correlation coefficients between the pilot ESDAM compared with the 3-d FR and the original FFQ for energy, absolute protein, total fat, and carbohydrate intake. Higher correlation coefficients were found between the pilot ESDAM and the FR compared with the FFQ and the FR for energy, fat, and carbohydrate intake (Figure 4).

**TABLE 2:** Convergent validity of pilot ESDAM compared with the FFQ and a 3-d food record (FR).

*N* = 27	FFQ		Pilot ESDAM		FR		Convergent validity: ESDAM vs. FR			
	Mean	SD	Mean	SD	Mean	SD	Mean difference	*P* value^1^	SCC	*P* value^2^
Energy (kcal)	1459	320	1759	359	1665	258	94	0.179	0.30	0.130
Absolute macronutrients										
Protein (g)	59	15	75	17	67	15	8	0.034	0.34	0.085
Total fat (g)	58	16	76	16	70	18	6	<0.001	0.24	0.228
Carbohydrate (g)	173	48	195	48	178	35	17	<0.001	0.47	0.014
Relative macronutrients										
Protein (%)	16	2	17	2	16	3	1	0.177	0.18	0.372
Total fat (%)	36	7	39	6	37	8	2	0.149	0.60	0.001
Carbohydrate (%)	47	8	44	6	43	7	1	0.139	0.68	<0.001

Abbreviation: ESDAM, experience sampling-based dietary assessment method; SCC, Spearman correlation coefficient.

1 *P* value of mean difference.

2 *P* value of SCC.

**FIGURE 5:**
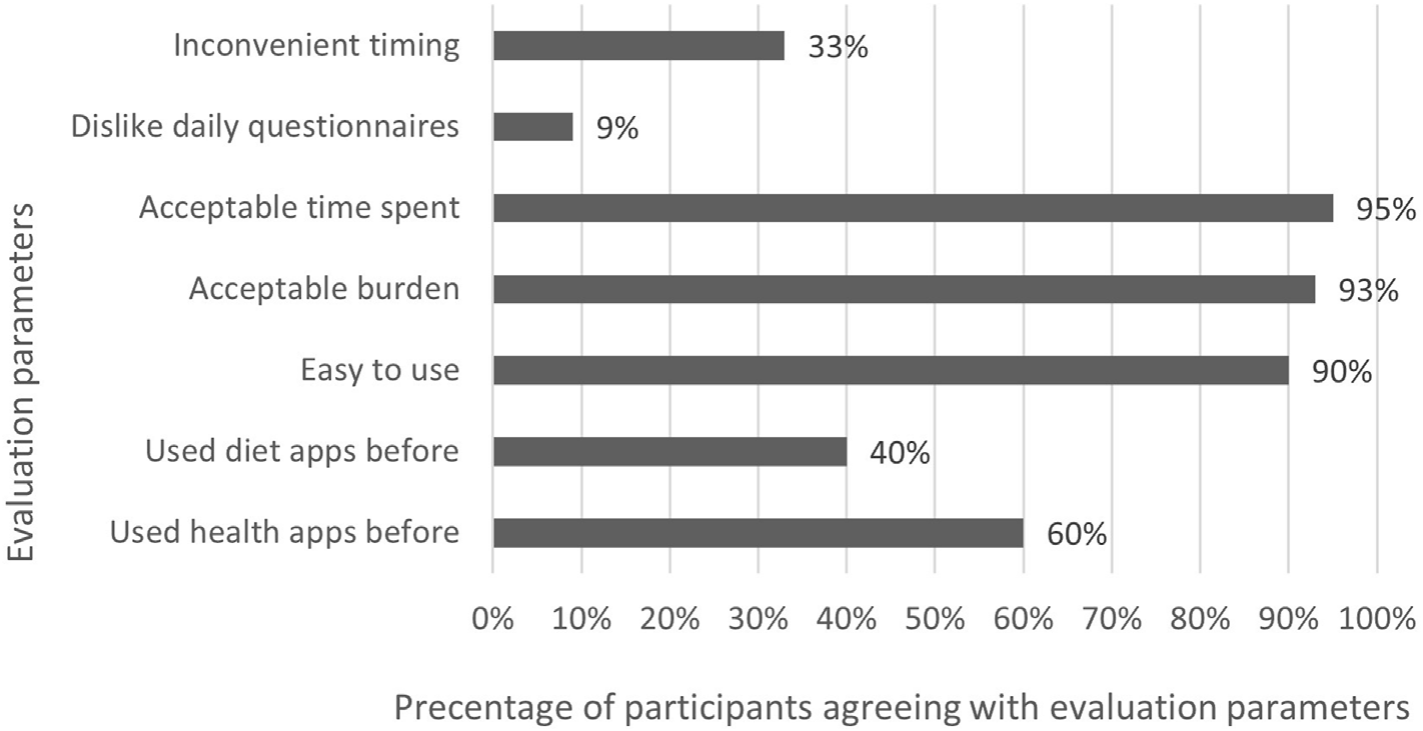
Results of the evaluation questionnaire on feasibility of the pilot ESDAM (n = 78*). ESDAM, experience sampling-based* dietary assessment method.

In total, 78 participants completed the pilot ESDAM and filled out the evaluation questionnaire. The results of the feasibility evaluation questionnaire are shown in Figure 5. More than 90% of participants indicated that the pilot ESDAM was easy to use, low burden, and the time spent to complete the pilot ESDAM questionnaire was acceptable. One-third (33%) found the timing of the pilot ESDAM questionnaire (19:00 with a response window until 23:00) as inconvenient.

### UX evaluation of the prototype ESDAM

The first round of the UX evaluation study included 10 participants of whom 9 participated in the evaluation interview. The mean age of the participants of the first UX round was 31 ± 10 y including 8 women, 1 men, and 1 unknown. The second round of the UX evaluation study included 10 participants with a mean age of 36 ±13 y including 6 women, 3 men, and 1 unknown. The results of the UX evaluation round 1 and round 2 are presented in Table 3. According to the feedback received following the first UX evaluation round, most participants (89%) had a positive experience using the first prototype ESDAM with responses including the method being rapid, easy, user friendly, and low burden. Most difficulties experienced in the first prototype ESDAM were concerning the response options. Difficulties with response options included uncertainty on the categorization of specific consumed foods into food group category response options (i.e., “Does plant-based milk also belong to the food category dairy products?,” “Does peanut butter need be added to the food category” “nuts and seeds” or to “sandwich spread”?). In addition, the estimation of portion sizes of consumed foods was reported as being difficult or taking more time to consider by 3 of 9 participants. However, the added examples of standard portion sizes in household measures within the response options were mentioned to be helpful. Following this feedback, the first prototype ESDAM was adapted regarding phrasing of certain food categories (i.e., “nuts and seeds” changed to “nuts and seeds including nut butter”). Additional examples of standard portion sizes in household measures were added to response options for specific food groups following the received feedback (i.e., weight of 1 apple was added as an example to response options of the food group fruit). The second round of UX evaluation in regard to the second prototype ESDAM revealed similar findings for overall app experience, and uncertainties in the questionnaire were reduced. Reported “ease of use” changed from 9.1 ± 0.6 (mean ± SD) in round 1 to 8.4 ± 0.9 (mean ± SD) in round 2 but was not statistically significantly different (*P* = 0.195). Timing and frequency of prompts, clarity of questions, flow, and time needed to respond remained the same for both round 1 and round 2 UX evaluations.

**TABLE 3:** UX evaluation results to evaluate the feasibility of the prototype ESDAM (*n* = 10 each round).

UX evaluation	Round 1	Round 2
Participants		
Responders	9	9
Nonresponders	1	1
Overall app experience		
Positive	8	9
Negative	0	0
Neutral	1	0
Ease of use (scale 0–10, 10 = very easy)		
Mean	9.1	8.4
Uncertainties in the questionnaire		
None	5	6
Attributing foods to correct food categories	2	1
Estimating portion sizes	1	1
Forgetting registration of beverages	1	1
Timing of prompts		
Acceptable	8	8
Inconvenient	1	1
Frequency of prompts		
Keep current frequency (3/d)	8	8
Higher frequency	1	1
Lower frequency	0	0
Clarity of questions		
Clearly formulated	8	8
Uncertainties	1	1
Clarity of response options		
Food category options		
No remarks	5	8
Uncertainties/difficulties	4	1
Portion size options		
No remarks	6	8
Uncertainties/difficulties	3	1
Flow to respond to questions		
Good or rapid	9	9
Not logical or slow	0	0
Time needed to respond		
Acceptable	9	9
Burdensome	0	0
Comparison with other diet tracking apps		
Did not use diet tracking apps before	5	8
Used diet tracking apps before	3	1

## Discussion

This manuscript describes the iterative development process of an ESDAM to assess habitual dietary intake. The pilot ESDAM could be considered an intermediate between the original FFQ and true ESM. Convergent validity assessment of the pilot ESDAM compared with a 3-d FR showed improved correlation coefficients compared with the FFQ. Therefore, ESM has shown potential as an alternative method for dietary assessment. Feasibility evaluation of the pilot ESDAM UX showed the pilot ESDAM to be rapid, easy to use, and low burden but questionnaire timing was found to be inconvenient. Evaluation of the prototypes of ESDAM showed good user acceptance and feasibility. The frequency and timing of prompts of the prototype ESDAM were considered acceptable by end-users, whereas ambiguity of content in response options were revealed.

Comparison of energy and macronutrient intake results between the pilot ESDAM and the FR and FFQ allowed us to assess convergent validity preliminary and, thus, the ability of the pilot ESDAM to enhance data accuracy. This preliminary evaluation of the convergent validity of the pilot ESDAM showed that the highest correlation coefficients were found between the pilot ESDAM and the FFQ for energy and all macronutrients (Figure 3). Because the pilot ESDAM is derived from the FFQ (i.e., same food groups and backend for nutrient calculation) it is expected to find high correlations due to correlated errors [29]. For this reason, a comparison between the pilot ESDAM and a reference method with fewer related errors, such as the FR, is considered more meaningful [3]. In addition, the FFQ was developed to measure habitual dietary intake of the past month and is, therefore, less suitable compared with a FR to assess the convergent validity of the ESDAM. In this sample (*n* = 27), higher correlation coefficients were found between the pilot ESDAM and the FR than between the FFQ and the FR for energy, fat, and carbohydrate intake. Moderate-to-good correlations were found with SCCs ranging from 0.18 for protein intake to 0.68 for relative carbohydrate intake, however, only statistically significant for absolute carbohydrate intake, relative fat, and relative carbohydrate intake. Post hoc power calculations showed that correlation coefficients of 0.30 can be detected with a 34% power and correlation coefficients of 0.50 can be detected with a 78% power for a sample size of 27 participants. Therefore, the results of the pilot evaluation should be interpreted with caution due to the small sample size (*n* = 27) and low power resulting from the low number of participants completing both the FFQ, 3-d FR, and ≥3 rounds of the ESDAM. Possibly the study design of the evaluation study including the duration of the study or multiple questionnaires yielded a high burden contributing to reporting fatigue. The evaluation questionnaire revealed the pilot ESDAM to be preferred over the FFQ due to low burden, ease of use, and limited time needed to complete. However, the timing of sending the pilot ESDAM (at 19:00) was often reported as inconvenient. Although the burden of the ESDAM was perceived as low, less than half of the participants completed the ESDAM for ≥3 of the 4 rounds possibly due to the inconvenient timing of the questionnaire.

UX evaluation revealed few improvements could be made regarding the labeling of food group categories in the response options and adding specific examples to aid estimation of portion sizes. Data saturation was reached relatively fast, following 2 rounds of UX evaluation, which could be explained by the fact that the questions and response options of the FFQ, from which the prototype ESDAM was developed, underwent content validity already [20]. Nevertheless, the results of the UX evaluation demonstrate the need for reassessment of content validity and UX when an existing validated questionnaire is adapted and implemented into a new methodology. As a result, the final ESDAM is a (near)–real-time smartphone-based dietary assessment method to assess habitual dietary intake considered being low burden, low cost, and user friendly.

To the best of our knowledge, the ESDAM is the first dietary assessment method based on experience sampling aimed to assess habitual dietary intake and developed based on an FFQ. In contrast, Lucassen et al. [19] developed a dietary assessment method named “Traqq” by applying EMA similarly to the methodology of a FR. Although a similar iterative development and evaluation process was followed, Traqq differs from ESDAM in the method of registering dietary intake. Although ESDAM collects dietary intake data at the meal level and food group level with multiple choice portion size options derived from an FFQ, Traqq has an open field search bar based on the national food consumption database allowing exact food consumption registration similar to a FR. Both Traqq and ESDAM show positive results regarding feasibility, whereas implementing a methodology that is substantially different from existing dietary assessment methods [19].

Although the ESDAM allows collecting near–real-time dietary intake data for longer periods of time without being burdensome, there are important limitations to consider. First, the ESDAM is a self-reported method as well and, therefore, will still be subjective to recall bias and reactivity bias albeit to a lesser extent due to the short recall period and near–real-time data collection at seemingly random moments. Eventually, the methodology of ESDAM shows features of both 24HRs and FFQs. It is questionable if a completely different methodology is possible for self-reported dietary assessment. The ESM can be considered a cross between 24HRs and FFQs. Second, according to our UX evaluation study, the random prompts were disliked by ∼10% of participants who preferred a food record approach over the ESDAM. Additionally, the frequency and timing of prompts might need to be tested for feasibility and adapted to the target population. Third, the use of a smartphone-based dietary assessment method requires a certain level of digital literacy. Therefore, the methodology might be less suitable for certain study populations. Lastly, the ESDAM is developed specifically for the Belgian dietary pattern and meal compositions. Application in other populations would require adaptations according to the specific food culture.

The strength of the ESDAM lies in the combined implementation of technology, by a smartphone-based tool, together with the new ESM and might, in this way, improve both feasibility and data accuracy. The results of the pilot ESDAM evaluation questionnaire show improved feasibility compared with the FFQ. An additional strength of the ESDAM is assessing dietary intake based on the type of meal that was consumed. In this way, the ESDAM is unique in not only assessing single nutrients but also assessing dietary intake at meal level allowing a different analysis of the dietary pattern. This meets the need for diet-health research where the focus has moved from assessing single nutrient intake in relation to health toward assessment of dietary intake at meal level and dietary pattern as a whole in relation to health [30]. Especially, because different dietary exposure variables might act synergistically and, therefore, analysis of dietary intake at the meal level or the dietary pattern as a whole might be more relevant in diet-health research. However, to evaluate if the ESDAM improves data accuracy by reducing measurement error through the seemingly random near–real-time data collection validity should be assessed against objective reference methods (i.e., doubly labeled water method, urinary nitrogen). Therefore, evaluation of the accuracy of the dietary data obtained by the ESDAM remains imperative for validation studies that will be undertaken as a next step.

In conclusion, the iterative development supported by literature, expert opinion, testing, and end-user evaluation has resulted in the first ESDAM to assess habitual dietary development from an FFQ. Based on our results, the ESDAM may be considered a low cost, low burden, and easy-to-use new dietary assessment tool to assess habitual dietary intake. In this way, ESM might advance the field of dietary assessment beyond traditional methods. However, validation studies, preferably including objective reference methods such as biomarkers (i.e., doubly labeled water, urinary nitrogen), will need to shed light on the quality and accuracy of dietary data obtained through the ESDAM.

## Author contributions

The authors’ responsibilities were as follows – JV, CM: designed the research; JV: conducted the research, analyzed the data, performed statistical tests, and wrote the paper; CM: had primary responsibility for the final content; and all authors read and approved the final manuscript.

## Funding

This research was funded by a PhD Fellowship Strategic Basic Research Grant (1S96721N) of the Research Foundation Flanders (FWO) and internal KU Leuven fund (C3/22/050). The funders had no involvement in any part of the research or report for publication.

## Data availability

Data described in the manuscript will be made available upon request pending application and approval.

## Conflict of interest

The authors report no conflicts of interest.
